# Administration of All-Trans Retinoic Acid to Pregnant Sows Improves the Developmental Defects of Hoxa1^−/−^ Fetal Pigs

**DOI:** 10.3389/fvets.2020.618660

**Published:** 2021-01-11

**Authors:** Haimei Zhou, Yixin Chen, Yongqiang Hu, Shan Gao, Wei Lu, Yuyong He

**Affiliations:** ^1^Jiangxi Province Key Laboratory of Animal Nutrition/Engineering Research Center of Feed Development, Jiangxi Agricultural University, Nanchang, China; ^2^Department of Animal Science, Jiangxi Agricultural Engineering College, Zhangshu, China

**Keywords:** all-trans retinoic acid, intrauterine growth retardation, ear defects, Hoxa1 mutation, fetal pig

## Abstract

Hoxa1 mutation adversely affect fetal pig development, but whether all-trans retinoic acid (ATRA) administration to Hoxa1^+/−^ pregnant sows can improve Hoxa1^−/−^ fetal pig development defects has not been reported. A total of 24 healthy Hoxa1^+/−^ sows were mated with a healthy Hoxa1^+/−^ boar and randomly assigned to one control group and nine experiment groups. ATRA was orally administered to pregnant sows at the doses of 0, 4, 5, or 6 mg/kg maternal body weight on 12, 13, and 14 days post coitum (dpc), respectively, and a total of 146 live piglets were delivered including 37 Hoxa1^−/−^ piglets and 109 non-Hoxa1^−/−^ piglets. Results indicated that Hoxa1^−/−^ piglets delivered by sows in control group had bilateral microtia, canal atresia and ear's internal defects, and had lower birth liveweight and external ear score than non-Hoxa1^−/−^ neonatal piglets (*P* < 0.05). Maternal administration with ATRA can effectively correct the development defects of Hoxa1^−/−^ fetal pigs, Hoxa1^−/−^ neonatal piglets delivered by sows administered ATRA at a dose of 4 mg/kg body weight on 14 dpc had higher birth liveweight (*P* > 0.05) and higher scores of external ear (*P* < 0.05) compared to Hoxa1^−/−^ neonatal piglets from the control group, but had no significantly difference in terms of birth liveweight and external ear integrity than non-Hoxa1^−/−^ piglets from the control group (*P* > 0.05). The time of ATRA administration significantly affected Hoxa1^−/−^ fetal development (*P* < 0.05). Administration of ATRA to Hoxa1^+/−^ pregnant sows at 4 mg/kg body weight on 14 dpc can effectively improve the birth liveweight and ear defects of Hoxa1^−/−^ piglets.

## Introduction

Gene mutations, nutrition imbalance or adverse maternal environments may lead to abnormal development or death of fetuses (1–3). For instance, hox cluster genes regulate the migration and differentiation of cranial neural crest cells (CNCC) and embryonic patterning, thereby vitally impacting fetal organogenesis during embryonic development (4, 5). Mutations of hox family genes may cause abnormal CNCC migration and in turn result in abnormal development of fetus (6–8). It has been shown that targeted Hoxa1 inactivation leads to severe reduction of rhombomere 4 (r4) and r5 and death shortly after birth (9). Gavalas et al. (10) found that the deletion of Hoxa1 and

Hoxb1 in mice significantly reduces the number of CNCC in the second branchial arch, eventually leading to malformation of the organs derived from the second branchial arch (10). In addition, Hoxa1 mutations can lead to auricle loss and external auditory canal damage (10, 11) and to cardio-cerebrovascular abnormalities (12, 13). Alasti et al. (14) found in Iranian humans families that a Q186K variant in hoxa2 leads to a phenotype of external ear malformation (14). Qiao et al. (15) firstly reported that the Hoxa1 mutation of g.50111251 G>TC results in abnormal auricle and external auditory canal, dyspnea, and even death in newborn piglets (15).

The initial transcription of hox family cluster genes requires the involvement of retinoic acid (RA) (16). RA is a physiologic active substance produced from the catalysis of vitamin A by retinol dehydrogenase and retinaldehyde dehydrogenase. RA can bind to the response elements of hox family genes and regulate the transcription and expression of hox family genes, including Hoxa1 (17–19). The concentration of RA required by different hox family genes for initial transcription varies (20–22). All-trans RA (ATRA) is one of the geometric isomers of RA (23) and is involved in cellular differentiation, morphogenesis and fetal growth (24). Molotkova et al. (25) found that RA regulates the differentiation and development of the posterior neuroectoderm of mouse embryos at gestational age 7.5–9.5 days (25). Improper RA supplementation leads to abnormal migration of CNCC and causes various degrees of external ear malformation (26–29), high dose RA maternal administration can produce malformations during organogenesis (26, 27), cleft palate can be developed when administrating retinoid acid to pregnant mice at gestational day 11 or day 17 with level of larger than 10 mg/kg body weight (30) and fetal inner ear dysmorphogenesis was also observed when gravid mice were administered RA with two consecutive doses of 25 mg/kg body weight (31). In contrast, proper nutrition supplementation before birth can correct outer ear malformation caused by congenital defects (32). Furthermore, Pasqualetti et al. (28) reported that low-dose exogenous RA (5 mg/kg BW) can repair inner ear defects caused by mutations in Hoxa1 of mice at 7.5–9.5 days of gestation, suggesting that RA can compensate for the functional loss caused by the Hoxa1 defect and this restorative effect is only effective at 8–8.75 days post coitum (28).

In the previous study we found that the Hoxa1 mutation of g.50111251 G>TC resulted in low birth liveweight, ear malformations, hearing impairment and dyspnea of Hoxa1^−/−^ neonatal piglets (15) and all Hoxa1^−/−^ new born piglets will die during suckling period, but no information has been reported regarding by using maternal ATRA administration to repair those defects in Hoxa1^−/−^ fetal pigs. The current study involved orally administering ATRA to Hoxa1^+/−^ pregnant sows to investigate the effects of ATRA on birth weight, ear development of Hoxa1^−/−^ neonatal piglets with an aim to determine the optimal dosage and time of ATRA administration for rescuing developmental defects of Hoxa1^−/−^ fetus.

## Materials and Methods

### Experimental Design

Twenty-four Hoxa1^+/−^ sows which derived from a Chinese Erhualian founder boar and Shaziling founder sow were selected and mated to a healthy Hoxa1^+/−^ boar, and then sows were randomly assigned to one control group (Group 0) and nine experimental groups (Group 1–9). ATRA was orally administered to sows as shown in Table 1. This experiment was complied with the policies of National Institutes of Health Guide for Care and Use of Laboratory Animals.

**Table 1:** Experimental design for the oral administration of all trans retinoic acid (ATRA) to Hoxa1^+/−^ pregnant sows.

**Treatment groups**	**Days post coitum for ATRA administration to sows**	**Dose of ATRA (mg/kg maternal body weight)**	**Numbers of sows**
Control group (G0)	0	0	6
Experimental group 1 (G1)	12	4	2
Experimental group 2 (G2)	12	5	2
Experimental group 3 (G3)	12	6	2
Experimental group 4 (G4)	13	4	2
Experimental group 5 (G5)	13	5	2
Experimental group 6 (G6)	13	6	2
Experimental group 7 (G7)	14	4	2
Experimental group 8 (G8)	14	5	2
Experimental group 9 (G9)	14	6	2

### Oral Administration of ATRA to Pregnant Sows

The amount of ATRA administered to each pregnant sow was calculated according to its body weight, and ATRA was dissolved in dimethyl sulfoxide (DMSO) at a ratio of 1:62.5 (w/w). The ATRA:DMSO mixture was diluted with 50 ml soybean oil and mixed with 0.5 kg basal diet and fed to sows on the scheduled date.

### Body Weight, Recording of External Ear Phenotype, and External Ear Scoring

**Figure 1:**
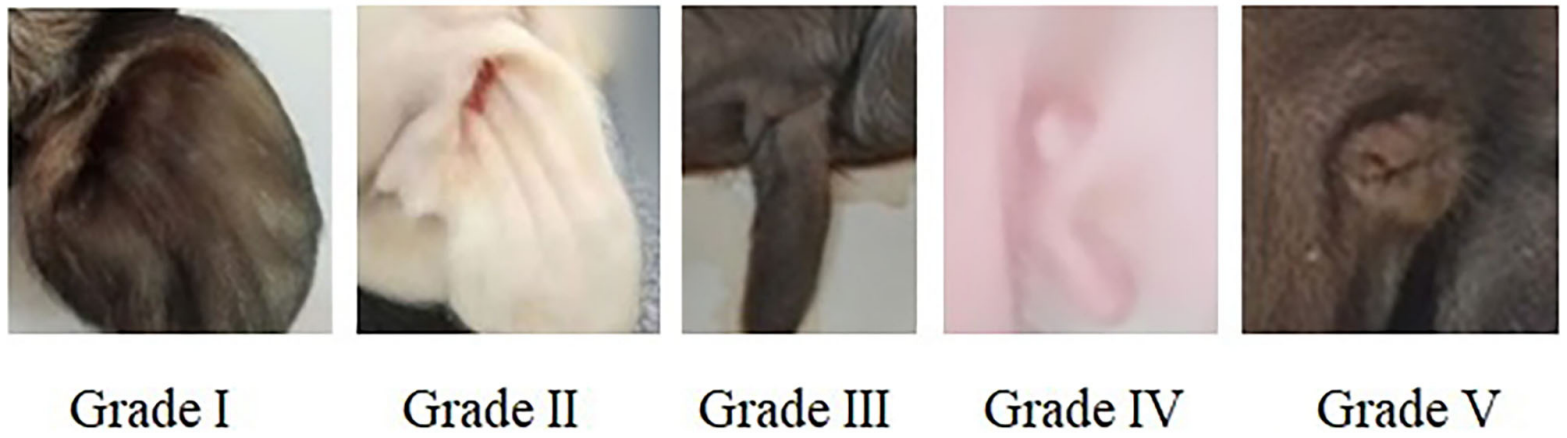
Score criteria for left and right external ear of neonatal piglets. Grade I: normal pinna with ear hole of external auditory canal, score 5. Grade II: small pinna with ear hole of external auditory canal, score 4. Grade III: strip pinna with atresia of external acoustic meatus, score 3. Grade IV: 2-3 “peanut” auricle with atresia of external acoustic meatus, score 2. Grade V: anotia, score 1.

All pregnant sows finished the farrowing within 20 days, newborn piglets were individually cleaned with dry towel and weighed immediately after birth. The external ear phenotype on the left and right side of each piglet was recorded and scored according to Figure 1.

### Sample Collection

Each piglet was dried with clean towel immediately after delivery, then several pieces of ear samples were cut off with ear notcher or tail samples were obtained by removing the end portion of tail with tail clipper from each piglet, ear or tail samples of each piglet were immediately transferred to Eppendorf tubes containing 75% alcohol and stored at 4°C in refrigerator for genotyping.

### Hoxa1- c.451 G>TC Genotyping

**Figure 2:**
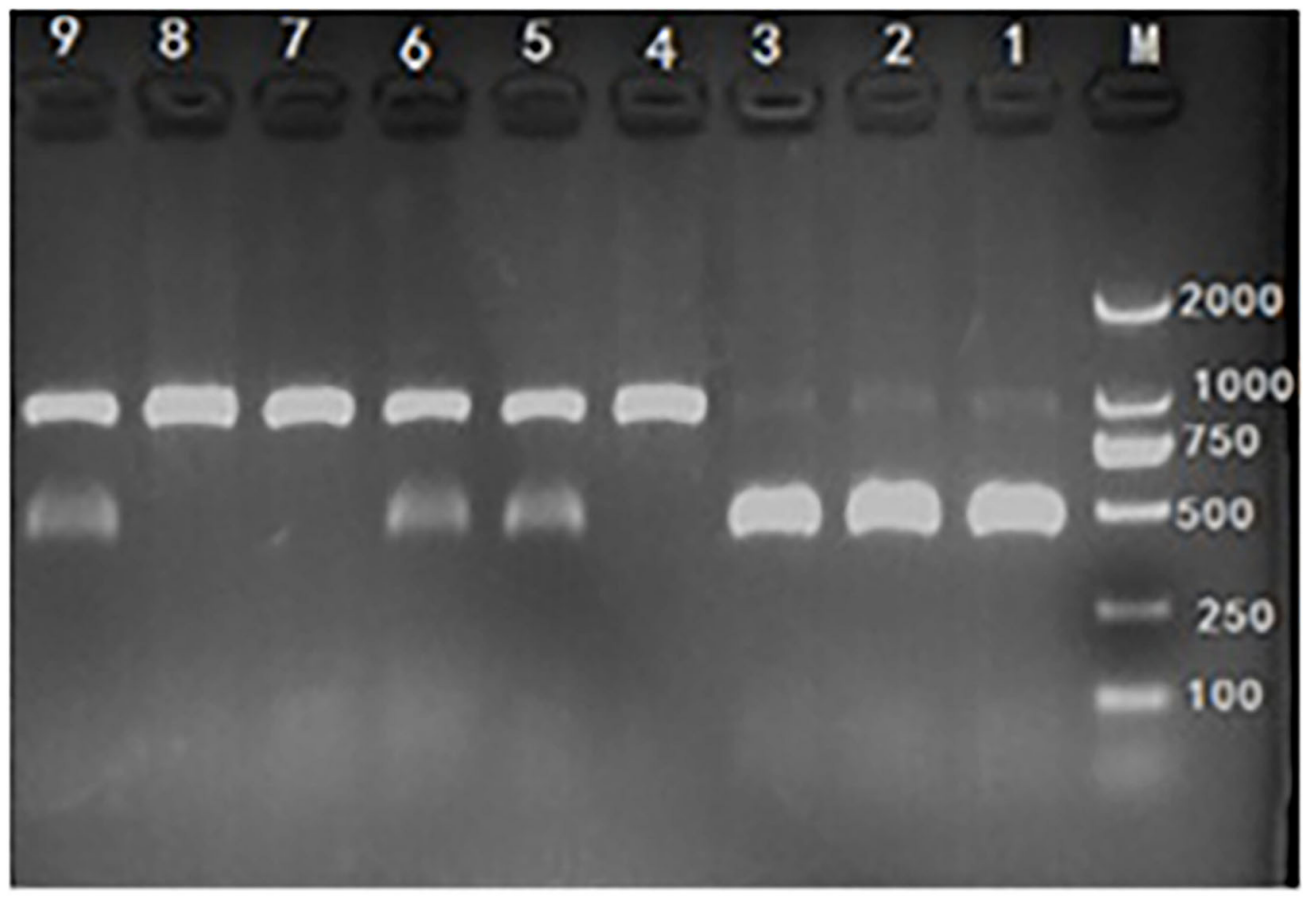
Hoxa1-c.451 G>TC genotyping by polymerase chain reaction-restriction fragment length polymorphism (PCR-RFLP) assay. M: marker. Lane 1, 2, and 3: mutant (Hoxa1^−/−^), abnormal external ear. Lane 4, 7, and 8: wild type (Hoxa1^+/+^), normal external ear. Lane 5, 6, and 9: heterozygote (Hoxa1^+/−^), normal external ear.

Genomic DNA from ear or tail tissues was extracted from each piglet using genomic DNA extraction Kit which was obtained from Genstar (Beijing, China). The concentration of extracted DNA was quantified and qualified using the A260/A280 ratio by NanoDrop ND-1000 UV spectrophotometer (NanoDrop Technologies, Rockland, DE) and genomic DNA was used as template for PCR amplification in 15-μl reaction mixtures containing 40 ng genomic DNA, 0.05 mM MgCl2, 0.2 μl 10 × Buffer, 0.4 mM dNTP, 1.0 U DNA polymerase, 20 pmol forward primer (5′-TGGACAATGCAAGAATGAGC-3′), and 20 pmol reverse primer (5′-CCCACGTCCTACTTCCAAAA-3′). PCR amplification was performed using the following conditions: 94°C/5 min followed by 30 cycles of 94°C, 30 s; 62°C, 30 s; and 72°C, 40 s; with a final elongation at 72°C for 8 min. Mutation from G to TC at base No. 451 of the Hoxa1 coding sequence was used for RFLP analysis with the following procedures (15): the PCR products were digested overnight at 37°C in 10 μl reaction mixtures (5 μl PCR product, 0.5 μl SmaI endonuclease, 1 μl 10 × T-buffer, 1 μl 0.1% BSA, and 2.5 μl H_2_O). The restriction fragments were stained with ethidium bromide, resolved on 1.5% agarose gels, and visualized under UV light. The wild-type allele (G) was represented by a restriction fragment of 891 bp and the mutant allele (TC) by two fragments of 438 bp and 453 bp, Hoxa1 was classified into one of three genotypes according to the bands produced by SmaI digestion (Figure 2).

### High-Resolution Computer Tomography (CT) Scan

In order to compare the differences in internal structures of ears of piglets, four neonatal piglets including two neonate piglets (one Hoxa1^−/−^ piglet and one Hoxa1^−/−^ piglet) from control group and two neonate piglets (one Hoxa1^−/−^ piglet with partially bilateral external defects of ears and one Hoxa1^−/−^ piglet with normally bilateral external structures of ears) from experimental group 7 were selected for high-resolution computer tomography scan. Imaging was performed on a Revolution ACTs 16-MDCT scanner (GE Healthcare) using the following parameters: electric voltage 120 kVp, scanning time 1.0 sec, slice thickness 1.25 mm, and pitch 0.938:1.

### Statistical Analyses

SPSS v. 17.0 software (IBM Corp., Armonk, NY, USA) was used for statistical analysis. All data were analyzed using two-way ANOVA (factors: date of ATRA administration and dose of ATRA) and Duncan's multiple comparison test was conducted to identify significant difference, differences were considered statistically significant if the *P*-value was ≤ 0.05.

## Results

### Administration of ATRA to Pregnant Hoxa1^+/-^ Sows Increased the Birth Liveweight of Hoxa1^-/-^ Piglets

As shown in Table 2, Hoxa1^−/−^ piglets in the control group had significantly lower average birth weights compared to the non-Hoxa1^−/−^ (Hoxa1^+/+^ and Hoxa1^+/−^) piglets (*P* < 0.05). While there was no significant difference in average birth weight of Hoxa1^−/−^ piglets among control group and experimental groups (*P* > 0.05) with an exception of G2, Hoxa1^−/−^ piglets delivered by sows of G2 had a significant lower average birth liveweight than Hoxa1^−/−^ piglets from G7 and G9, respectively (*P* < 0.05) but had no significant lower average birth liveweight compared to Hoxa1^−/−^ piglets from other groups (*P* > 0.05). The average birth weight of the Hoxa1^−/−^ piglets born from sows treated with ATRA on 14 dpc at doses of 4 mg/kg body weight and 6 mg/kg body weight were numerically higher than those born from control group and the other experimental groups (*P* > 0.05). In addition, the average birth liveweight of non-Hoxa1^−/−^ piglets delivered by sows from control group was significantly higher than that of non-Hoxa1^−/−^ piglets born by sows from experimental groups (*P* < 0.05). The Days of ATRA administration, dose of ATRA and the interaction of the days and dosage had significant effect on the birth liveweight of Hoxa1^−/−^ piglets (*P* < 0.01).

**Table 2:** Effect of oral administration of all-trans retinoic acid (ATRA) to pregnant Hoxa1^+/−^ sows on birth live weight of Hoxa1^−/−^ piglets.

**Genotype of piglets**	**Treatment groups**	**Number of piglets**	**Average birth liveweight of piglets (kg)**
Non-Hoxa1^−/−^	G0	20	1.22^a^
	G1-9	89	0.92^b^
Hoxa1^−/−^	G0	5	0.80^bc^
	G1	3	0.72^bc^
	G2	4	0.68^c^
	G3	4	0.85^bc^
	G4	3	0.70^bc^
	G5	3	0.74^bc^
	G6	3	0.89^bc^
	G7	5	0.94^ab^
	G8	3	0.88^bc^
	G9	4	0.95^ab^
SEM			0.02
*P*-value	Days of ATRA administration		0.00
	Dose of ATRA		0.00
	Days × Dosage		0.00

G0: 0 mg ATRA; G1: 12 dpc + 4 mg ATRA; G2: 12 dpc + 5 mg ATRA; G3: 12 dpc + 6 mg ATRA; G4: 13 dpc + 4 mg ATRA; G5: 13 dpc + 5 mg ATRA; G6: 13 dpc + 6 mg ATRA; G7: 14 dpc + 4 mg ATRA; G8: 14 dpc + 5 mg ATRA; G9: 14 dpc + 6 mg ATRA. Means within a column followed by the same lower case letter do not differ significantly (P > 0.05). Means within a column followed by the different lower case letter differ significantly (P <0.05).

### Maternal Administration With ATRA Repaired the External Defects of Ears of Hoxa1^-/-^ Piglets

**Figure 3:**
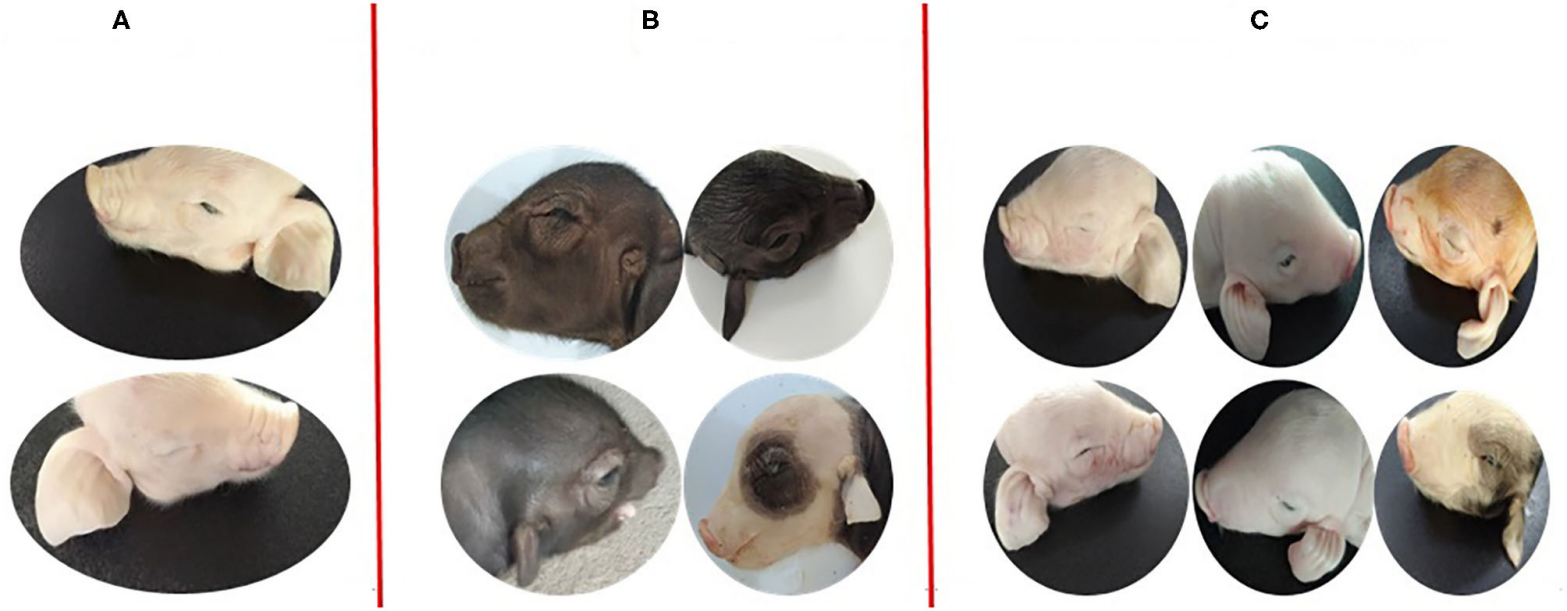
Phenotypic comparison of the differences between normal and defected external ears and the differences of repairing defected external ears with ATRA. **(A)** One non-Hoxa1^−/−^ neonatal piglet from control groups with normal bilateral external ears. **(B)** The representatives of Hoxa1^−/−^ neonatal piglets from control groups with different defected external ears **(C)** four Hoxa1^−/−^ neonatal piglets from G7: the left one with the completely repaired bilateral external ears; the middle one and the right two with the partially repaired external ears.

All non-Hoxa1^−/−^ (Hoxa1^+/+^ and Hoxa1^+/−^) new born piglets delivered by sows in control and experimental groups had normally bilateral external ears (normal pinna and external auditory meatus), one of the non-Hoxa1^−/−^ piglets was selected as the representative and presented its external bilateral ears in Figure 3A. All new born Hoxa1^−/−^ piglets in control group had defected bilateral external ears (bilateral microtia and atresia of external auditory meatus) and the defected external ears of four piglets were selected as the representatives and showed as Figure 3B. The deformed external ears of Hoxa1^−/−^ piglets from experimental groups were partially or completely repaired by maternal ATRA administration, four Hoxa1^−/−^ piglets from experimental group 7 were selected to display the effect of repairing defected external ears with maternal ATRA administration including one Hoxa1^−/−^ piglet with completely repaired bilateral external ears (left of Figure 3C) and three Hoxa1^−/−^ piglets with partially repaired external ears (middle and right of Figure 3C). Scoring of outer ears of each new born piglet was performed according to Figure 1 and the data are presented in Table 3. All non-Hoxa1^−/−^ piglets either from control group or different experimental groups were delivered with normal pinna and ear hole of external auditory meatus on each side of the head and had a score of 5, Hoxa1^−/−^ piglets in the control group were born with pinna defects and no ear hole of external auditory meatus on each side of the head and had lower score than new born non-Hoxa1^−/−^ piglets had (*P* < 0.05). Hoxa1^−/−^ piglets in the experimental groups were delivered with partial or no defects of pinna and ear hole on each side of the head and had higher score than Hoxa1^−/−^ newborn piglets in the control group with an exception in experimental group 5. One Hoxa1^−/−^ piglet that delivered by a sow in experimental group 7 had the normal pinna and ear hole of external auditory meatus on each side of the head and had the same score as non-Hoxa1^−/−^ piglets had. The date of ATRA administration had significant effect on the repair of outer ear defects of Hoxa1^−/−^ piglets (*P* < 0.01).

**Table 3:** Effect of oral administration of all-trans retinoic acid (ATRA) to pregnant Hoxa1^+/−^ sows on external Ear development of Hoxa1^−/−^ piglets.

**Genotype of piglets**	**Treatment groups**	**Number of piglets**	**Score of left ear of piglets**	**Score of right ear of piglets**
Non-Hoxa1^−/−^	G0	20	5.00^a^	5.00^a^
	G1-9	89	5.00^a^	5.00^a^
Hoxa1^−/−^	G0	5	1.67^c^	1.83^de^
	G1	3	3.00^abc^	3.00^bcd^
	G2	4	3.00^bc^	3.50^abc^
	G3	4	2.00^c^	2.50^bcde^
	G4	3	3.00^bc^	3.00^bcd^
	G5	3	1.50^c^	1.00^e^
	G6	3	2.33^bc^	2.67^bcde^
	G7	5	3.80^ab^	3.80^ab^
	G8	3	2.50^bc^	2.00^cde^
	G9	4	2.75^bc^	3.00^bcd^
SEM			0.21	0.21
*P*-value	Days of ATRA administration		0.00	0.00
	Dose of ATRA		0.46	0.36
	Days × Dosage		0.19	0.29

Means within a column followed by the same lower case letter do not differ significantly (P > 0.05). Means within a column followed by the different lower case letter differ significantly (P <0.05).

### Maternal Administration With ATRA Improved the Internal Defects of Ears of Hoxa1^-/-^ Fetal Piglets

**Figure 4:**
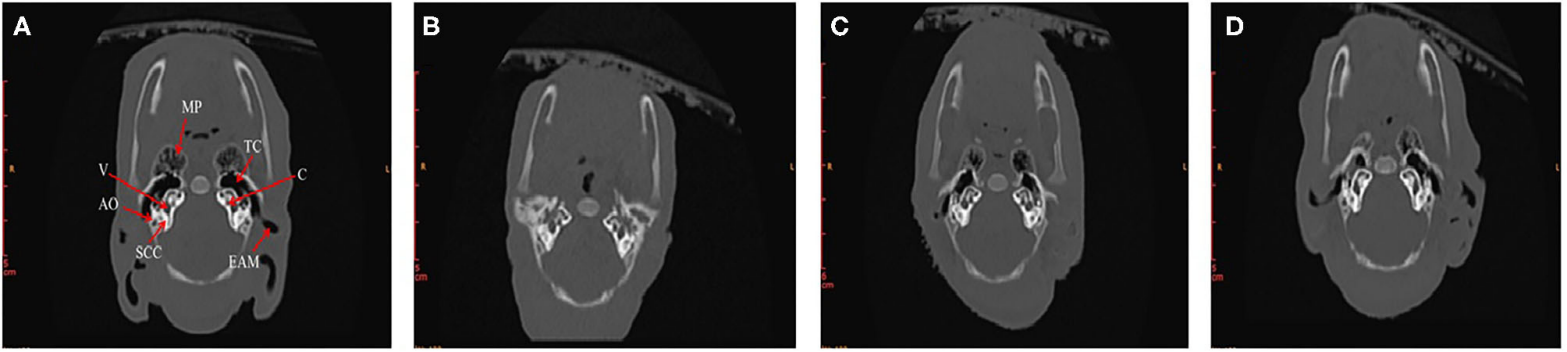
High-resolution CT imaging of internal structures of ears. **(A)** The non-Hoxa1^−/−^ piglet of Figure 3A had the normal internal structures of ears on each side of the head. **(B)** The Hoxa1^−/−^ piglet of Figure 3B had the complete absence of EAM, TC and MP. **(C)** The Hoxa1^−/−^ piglet from the middle of Figure 3C had the partially repaired internal structures of ears. **(D)** The Hoxa1^−/−^ piglet from the left of Figure 3C had the same normal internal structures of ears as the non-Hoxa1^−/−^ piglet of Figure 3A had. EAM, external auditory meatus. MP, mastoid process. TC, tympanic cavity. AO, auditory ossicle. SCC, semicircular canals. V, vestibule. C, cochlea.

In order to find out what are the differences in internal structures of ears among piglets that presented in Figure 3, we selected the non-Hoxa1^−/−^ piglet of Figure 3A, one of the Hoxa1^−/−^ piglet from Figure 3B (randomly), the Hoxa1^−/−^ piglet with completely repaired bilateral external ears (left of Figure 3C) and the Hoxa1^−/−^ piglet with partially repaired bilateral external ears (middle of the Figure 3C) to scan their internal structures of ears. Figure 4A exhibits that the non-Hoxa1^−/−^ piglet of Figure 3A had the normal internal structures of ears on each side including external auditory meatus (EAM), auditory ossicles (AO), semicircular canal (SCC), vestibule, cochlea, and tympanic cavity (TC). Mutation of Hoxa1 caused not only the defected external ears but also the loss of internal structures of ears, because one of the Hoxa1^−/−^ piglet from Figure 3B showed the losses of EAM, TC and MP and the deformed AO, SCC, vestibule and cochlea (Figure 4B). Maternal administration with ATRA in G7 was the most effective in repairing the internal defects of ears of Hoxa1^−/−^ piglets, because the internal defects of ears of the Hoxa1^−/−^ piglet from the middle of Figure 3C were partially repaired (Figure 4C), and the Hoxa1^−/−^ piglet from the left of Figure 3C had the normal internal structures of ears as newborn non-Hoxa1^−/−^ piglets had (Figure 4D).

## Discussion

Hoxa1 and ATRA are essential for the normal development of ears and other body tissues of vertebrate embryos (12, 33, 34). Both Hoxa1 mutations and ATRA deficiency can cause abnormal ear development of fetuses and newborn infants with ear abnormalities being associated with intrauterine growth retardation (IUGR) (8), including the low head growth and low body weight of fetuses (12, 35, 36). In the present study, all neonatal Hoxa1^−/−^ piglets born from sows in the control group presented little or no response to sound stimuli as our previous report (15) and had significantly lower live weight at birth than non-Hoxa1^−/−^ littermate piglets (*P* < 0.05). László et al. (8) reported that adequate external sound stimuli is an effective method in promoting normal fetal growth during the last trimester of pregnancy and hearing loss of fetuses is associated with low intrauterine growth (8). After oral administration of ATRA to Hoxa1^+/−^ pregnant sows at a dose of 4 mg/kg body weight on 14 dpc, their offsprings with Hoxa1^−/−^ gene had more intact auricle and external auditory meatus and higher birth live weight than offsprings with Hoxa1^−/−^ gene in control group.

The external ear develops from the mesenchyme of the first and second pharyngeal arches and the identity of these pharyngeal arches is determined by rhombomere segmentation (37, 38). In vertebrate embryos, RA is synthesized by retinaldehyde dehydrogenase 2 (Raldh2) in mesoderm (17) and binds to hox nuclear receptors to activate anteroposterior (AP)-restricted Hox expression patterns and rhombomere segmentation in the hindbrain (13, 39, 40). However, Raldh2 mesodermal expression is under direct transcriptional control of the Hoxa1-Pbx1/2-Meis2 complex 9, when a Hoxa1 mutation occurs, the function of the Hoxa1-Pbx1/2-Meis2 complex can be affected, leading to a reduction in the production of endogenous RA, abnormal outer ear development and ATRA deficiency (41). Hoxa1^−/−^ mice showed abnormalities in hindbrain rhombomere segmentation and neural crest migration and presented with external ear defects (9, 42, 43). Administration of exogenous RA to pregnant mice at a dose of 2.5 mg/kg on day E7.5 or E8.5 effectively rescues the Hoxa1 mutant mice from inner ear defects (28, 41). Qiao et al. (15) firstly reported that newborn piglets develop unilateral or bilateral microtia or anotia when a c.451 G>TC mutation occurs in Hoxa1 piglets (15). Results from the current study showed that the administration of ATRA to pregnant Hoxa1^+/−^ sows at doses of 4, 5, and 6 mg/kg body weight improved the development of ears of Hoxa1^−/−^ fetuses, because maternal administration with ATRA partially or completely repaired the external defects of ears of Hoxa1^−/−^ fetal piglets, and the most effective regimen for repairing ear defects of Hoxa1^−/−^ fetuses was to administer exogenous ATRA to pregnant Hoxa1^+/−^ sows at a dose of 4 mg/kg maternal body weight on 14 dpc, because neonatal Hoxa1^−/−^ piglets in this group had the highest external ear scores and intact internal ear structures compared to those in the other experimental groups. One Hoxa1^−/−^ piglet delivered by a sow given 4 mg/kg exogenous ATRA on 14 dpc developed normal structures of external and internal ears, the possible explanation might be that this piglet received more exogenous maternal ATRA via the placenta than the other fetuses.

## Conclusions

Hoxa1 mutations produce low birth liveweight and deformed ears of neonatal piglets. Administration of ATRA to Hoxa1^+/−^ pregnant sows on 14 dpc at a dose of 4 mg/kg can improve birth liveweight and ear defects of Hoxa1^−/−^ neonatal piglets. The findings of the present study can provide some useful information for how to repair fetal developmental defects with maternal treatment in the animal healthy production of gene mutant fetuses and in the prevention of human genetic ear disease during pregnancy instead of surgical repair after birth.

## Data Availability Statement

The original contributions presented in the study are included in the article/supplementary materials, further inquiries can be directed to the corresponding author/s.

## Ethics Statement

The animal study was reviewed and approved by the Ethics Committee for Animal Experimentation of Jiangxi Agricultural University. Written informed consent was obtained from the owners for the participation of their animals in this study.

## Author Contributions

WL and YHe: conception and experimental design. HZ, YC, YHu, and SG: investigation. HZ and YC: data analysis. YHe and HZ: manuscript preparation. All authors have read and agreed to the published version of the manuscript.

## Conflict of Interest

The authors declare that the research was conducted in the absence of any commercial or financial relationships that could be construed as a potential conflict of interest.

## Abbreviations

ATRA: all-trans retinoic acid
dpc: days post coitum
CNCC: cranial neural crest cells
RA: retinoic acid
DMSO: dimethyl sulfoxide
DNA: deoxyribonucleic acid
UV: ultraviolet
dNTP: deoxy-ribonucleoside triphosphate
PCR: polymerase chain reaction
RFLP: restriction fragment length polymorphism
CT: computer tomography
MDCT: multidetector computer tomography
ANOVA: analysis of variance
EAM: external auditory meatus
MP: mastoid process
AO: auditory ossicles
SCC: semicircular canal
TC: tympanic cavity
IUGR: intrauterine growth retardation
AP: anteroposterior.

## References

[1] Bischof JM Stewart CL Wevrick R Inactivation of the mouse Magel2 gene results in growth abnormalities similar to Prader-Willi syndrome. Hum Mol Genet. (2017) 16:2713–9. 10.1093/hmg/ddm225 17728320

[2] Schaaf CP Gonzalez-Garay ML Xia F Potocki L Gripp KW Zhang BL. Truncating mutations of MAGEL2 cause Prader-Willi phenotypes and autism. Nat Genet. (2013) 45:1405–8. 10.1038/ng.2776 24076603 PMC3819162

[3] Guo W Nie YL Yan ZQ Zhu XH Wang YQ Guan S. Genetic testing and pgd for unexplained recurrent fetal malformations with magel2 gene mutation. Sci China Life Sci. (2019) 62:1–9. 10.1007/s11427-019-9541-0 31152388

[4] Cillo C Cantile M Faiella A Boncinelli E. Homeobox genes in normal and malignant cells. J Cell Physiol. (2001) 188:161–9. 10.1002/jcp.1115 11424082

[5] Garcia-Fernández J. The genesis and evolution of homeobox gene clusters. Nat Rev Genet. (2005) 6:881–92. 10.1038/nrg1723 16341069

[6] Chisaka O Musci TS Capecchi MR. Developmental defects of the ear, cranial nerves and hindbrain resulting from targeted disruption of the mouse homeobox gene Hox-#150;1.6. Nature. (1992) 355:516–20. 10.1038/355516a0 1346922

[7] Watt FM Hogan BLM. Out of Eden: stem cells and their niches. Science. (2000) 287:1427–30. 10.1126/science.287.5457.1427 10688781

[8] László P Ferenc B Czeizel AE. Maternal characteristics and birth outcomes of pregnant women who had offspring with congenital ear abnormalities - a population-based case control study. J Matern Fetal Neonatal Med. (2011) 24:1107–14. 10.3109/14767058.2010.545924 21401310

[9] Mark M Lufkin T Vonesch JL Ruberte E Olivo JC Dollé P. Two rhombomeres are altered in hoxa-1 mutant mice. Development. (1993) 119:319–38. 8287791 10.1242/dev.119.2.319

[10] Gavalas A Trainor P Ariza-Mcnaughton L Krumlauf R. Synergy between Hoxa1 and Hoxb1: the relationship between arch patterning and the generation of cranial neural crest. Development. (2001) 128:3017–27. 11532923 10.1242/dev.128.15.3017

[11] Rossel M Capecchi MR. Mice mutant for both Hoxa1 and Hoxb1 show extensive remodeling of the hindbrain and defects in craniofacial development. Development. (1999) 126:5027–40. 10529420 10.1242/dev.126.22.5027

[12] Tischfield MA Bosley TM Salih MAM Alorainy IA Sener EC Nester MJ. Homozygous Hoxa1 mutations disrupt human brainstem, inner ear, cardiovascular and cognitive development. Nat Genet. (2005) 37:1035–7. 10.1038/ng1636 16155570

[13] Makki N Capecchi MR. Hoxa1 lineage tracing indicates a direct role for Hoxa1 in the development of the inner ear, the heart, and the third rhombomere. Dev Biol. (2010) 341:499–509. 10.1016/j.ydbio.2010.02.014 20171203 PMC2862106

[14] Alasti F Sadeghi A Sanati MH Farhadi M Stollar E Somers T. A mutation in Hoxa2 is responsible for autosomal-recessive microtia in an Iranian family. Am J Hum Genet. (2008) 82:982–91. 10.1016/j.ajhg.2008.02.015 18394579 PMC2427268

[15] Qiao RM He YY Pan B Xiao SJ Zhang XF Li J. Understanding the molecular mechanisms of human microtia via a pig model of Hoxa1 syndrome. Dis Model Mech. (2015) 8:611–22. 10.1242/dmm.018291 26035869 PMC4457031

[16] Martinez-Ceballos E Chambon P Gudas LJ Differences in gene expression between wild type and HOXA1 knockout embryonic stem cells after retinoic acid treatment or leukemia inhibitory factor (LIF) removal. J Biol Chem. (2015) 280:16484–98. 10.1074/jbc.M414397200 15722554

[17] Duester G. Retinoic acid synthesis and signaling during early organogenesis. Cell. (2008) 134:921–31. 10.1016/j.cell.2008.09.002 18805086 PMC2632951

[18] Delacroix L Moutier E Altobelli G Gras SL Poch O Choukrallah MA. Cell-specific interaction of retinoic acid receptors with target genes in mouse embryonic fibroblasts and embryonic stem cells. Mol Cell Biol. (2010) 30:231–344. 10.1128/MCB.00756-09 19884340 PMC2798310

[19] Lee LMY Leung CY Tang WWC Choi HL Leung YC McCaffery PJ. A paradoxical teratogenic mechanism for retinoic acid. PNAS. (2012) 109:13668–73. 10.1073/pnas.1200872109 22869719 PMC3427051

[20] Boncinelli E Simeone A Acampora D Mavilio F. HOX gene activation by retinoic acid. Trends Genet. (1991) 7:329–34. 10.1016/0168-9525(91)90423-N 1685814

[21] Wendling O Dennefeld C Chambon P Mark M. Retinoid signaling is essential for patterning the endoderm of the third and fourth pharyngeal arches. Development. (2001) 127:1553–62. 10725232 10.1242/dev.127.8.1553

[22] Gudas LJ Wagner JA. Retinoids regulate stem cell differentiation. J Cell Physiol. (2011) 26:322–30. 10.1002/jcp.22417 20836077 PMC3315372

[23] Blomhoff R Blomhoff HK. Overview of retinoid metabolism and function. J Neurobiol. (2006) 66:606–30. 10.1002/neu.20242 16688755

[24] Mark M Ghyselinck NB Chambon P. Function of retinoid nuclear receptors: lessons from genetic and pharmacological dissections of the retinoic acid signaling pathway during mouse embryogenesis. Annu Rev Pharmacol Toxicol. (2006) 46:451–80. 10.1146/annurev.pharmtox.46.120604.141156 16402912

[25] Molotkova N Molotkov A Duester G. Role of retinoic acid during forebrain development begins late when Raldh3 generates retinoic acid in the ventral subventricular zone. Dev Biol. (2007) 303:601–10. 10.1016/j.ydbio.2006.11.035 17207476 PMC1994967

[26] Kessel M Gruss AP. Homeotic transformations of murine vertebrae and concomitant alteration of codes induced by retinoic acid. Cell. (1991) 67:89–104. 10.1016/0092-8674(91)90574-I 1680565

[27] Wei X Makori N Peterson PE Hummler H Hendrickx AG. Pathogenesis of retinoic acid-induced ear malformations in primate model. Teratology. (1999) 60:83–92. 10.1002/(SICI)1096-9926(199908)60:2<83::AID-TERA12>3.0.CO;2-O 10440780

[28] Pasqualetti M Neun R Davenne M Rijli FM. Retinoic acid rescues inner ear defects in Hoxa1 deficient mice. Nat Genet. (2001) 29:34–9. 10.1038/ng702 11528388

[29] Romand R Dollé P Hashino E. Retinoid signaling in inner ear development. J Neurobiol. (2006) 66:687–704. 10.1002/neu.20244 16688766

[30] Hansen DK Laborde JB Wall KS Hinson WG Pipkin JL Shaddock J. Dose-response of retinoic acid induced stress protein synthesis and teratogenesis in mice. Reprod Toxicol. (2001) 15:31–41. 10.1016/S0890-6238(00)00118-0 11137376

[31] Frenz DA Liu W Cvekl A Xie Q Wassef L Quadro L. Retinoid signaling in inner ear development: a “Goldilocks” phenomenon. Am J Med Genet. (2010) 152A:2947–61. 10.1002/ajmg.a.33670 21108385 PMC3057869

[32] Chen WH Morriss-Kay GM Copp AJ. Genesis and prevention of spinal neural tube defects in the curly tail mutant mouse: involvement of retinoic acid and its nuclear receptors RAR-beta and RAR-gamma. Development. (1995) 121:681–91. 7720576 10.1242/dev.121.3.681

[33] Cunningham TJ Duester G. Mechanisms of retinoic acid signalling and its roles in organ and limb development. Nat Rev Mol Cell Biol. (2015) 16:110–23. 10.1038/nrm3932 25560970 PMC4636111

[34] Stefanovic S Zaffran S. Mechanisms of retinoic acid signaling during cardiogenesis. Mech Dev. (2016) 143:9–19. 10.1016/j.mod.2016.12.002 28007475

[35] Muscarella LA Guarnieri V Sacco R Militerni R Bravaccio C Trillo S. Hoxa1 gene variants influence head growth rates in humans. Am J Med Genet B Neuropsychiatr Genet. (2007) 144B:388–90. 10.1002/ajmg.b.30469 17171652

[36] De Kumar B Parker HJ Paulson A Parrish ME Zeitlinger J Krumlauf R. Hoxa1 targets signaling pathways during neural differentiation of ES cells and mouse embryogenesis. Dev Biol. (2017) 432:151–64. 10.1016/j.ydbio.2017.09.033 28982536

[37] Glover JC Jean-Sébastien R Rijli FM. Retinoic acid and hindbrain patterning. J Neurobiol. (2006) 66:705–25. 10.1002/neu.20272 16688767

[38] Maden M. Retinoic acid in the development, regeneration and maintenance of the nervous system. Nat Rev Neurosci. (2007) 8:755–65. 10.1038/nrn2212 17882253

[39] Lohnes D Kastner P Dierich A Mark M LeMeur M Chambon P. Function of retinoic acid receptor gamma in the mouse. Cell. (1993) 73:643–58. 10.1016/0092-8674(93)90246-M 8388780

[40] Mark M Ghyselinck NB Chambon P. Function of retinoic acid receptors during embryonic development. Nucl Recept Signal. (2009) 7:e002. 10.1621/nrs.07002 19381305 PMC2670431

[41] Vitobello A Ferretti E Lampe X Vilain N Ducret S Ori M. Hox and Pbx factors control retinoic acid synthesis during hindbrain segmentation. Dev Cell. (2011) 20:469–82. 10.1016/j.devcel.2011.03.011 21497760 PMC3677862

[42] Lufkin T Dierich A Lemeur M Mark M Chambon P. Disruption of the Hox-1.6 homeobox gene results in defects in a region corresponding to its rostral domain of expression. Cell. (1991) 66:1105–19. 10.1016/0092-8674(91)90034-V 1680563

[43] Gavalas A Studer M Lumsden A Rijli FM Krumlauf R Chambon P. Hoxa1 and Hoxb1 synergize in patterning the hindbrain, cranial nerves and second pharyngeal arch. Development. (1998) 125:1123–36. 9463359 10.1242/dev.125.6.1123

